# Should PNPO Deficiency Be Treated In Utero? Clinical Findings From Prenatal Pyridoxine Therapy

**DOI:** 10.1002/jmd2.70086

**Published:** 2026-04-20

**Authors:** Chloé de Puyraimond, Samia Pichard, Muriel Girard, Julian Delanne, Laurence Faivre, Apolline Imbard, Amandine Baurand, Jean‐François Benoist, Philippa B. Mills, Peter T. Clayton, Manuel Schiff

**Affiliations:** ^1^ Reference Center for Inherited Metabolic Diseases, Hôpital Necker‐Enfants Malades APHP and Université Paris Cité Paris France; ^2^ Hepatology Department, Hôpital Necker‐Enfants Malades APHP and Université Paris Cité Paris France; ^3^ Université Bourgogne Europe, CHU Dijon Bourgogne, Inserm, CTM UMR1231, Centre de génétique Dijon France; ^4^ Metabolic Biochemistry, Hôpital Necker‐Enfants Malades, APHP Paris France; ^5^ Département Médicaments et Technologies pour la Santé, MetaboHUB Université Paris‐Saclay, CEA Gif‐sur‐Yvette France; ^6^ Inborn Errors of Metabolism, Genetics and Genomic Medicine UCL Great Ormond Street Institute of Child Health London UK; ^7^ Inserm UMRS_1163, Institut Imagine Paris France

**Keywords:** in utero treatment, PLP, PNPO deficiency, prenatal therapy, pyridoxal‐5′‐phosphate, pyridoxine

## Abstract

Pyridox(am)ine‐5′‐phosphate oxidase (PNPO) deficiency is characterized by early‐onset epileptic encephalopathy refractory to standard antiseizure medications. It is caused by variants in the *PNPO* gene, resulting in deficient PNPO enzyme activity, which normally converts pyridoxine‐5′‐phosphate and pyridoxamine‐5′‐phosphate (two vitamers of vitamin B6) into the active cofactor, pyridoxal‐5′‐phosphate (PLP). Treatment relies on PLP or pyridoxine in some cases. Long‐term outcomes remain suboptimal. We describe two cases of unrelated children treated with vitamin B6 (pyridoxine) in utero: one with a confirmed prenatal PNPO diagnosis and one at risk due to family history but ultimately unaffected. In utero pyridoxine, combined with early postnatal PLP treatment, allowed excellent seizure control and normal neurodevelopment in the long term. Notably, our first patient, treated from birth, is now 10 years old, representing the oldest reported PNPO‐deficient individual treated from birth. Case 2 highlights the safety of prenatal B6 supplementation even in unaffected fetuses. These observations support prenatal pyridoxine supplementation as a safe and potentially beneficial strategy in at‐risk PNPO pregnancies.

## Take‐Home Message

In a context of family history with PNPO deficiency, in utero pyridoxine treatment combined with immediate postnatal PLP or pyridoxine treatment is a safe strategy to allow a good control of seizures and a normal neurodevelopment in the long term.

## 1 Introduction

Pyridox(am)ine‐5′‐phosphate oxidase (PNPO) deficiency is characterized by early‐onset epilepsy refractory to usual antiseizure medication. It is caused by biallelic variants in the *PNPO* gene leading to a deficiency of the enzyme PNPO, which converts two vitamers of vitamin B6, pyridoxine‐5′‐phosphate and pyridoxamine‐5′‐phosphate, into their active form pyridoxal‐5′‐phosphate (PLP). Treatment of PNPO deficiency primarily relies on PLP. Although earlier reports suggested that up to 40% of patients may respond to pyridoxine [1, 2], more recent data indicate that sustained pyridoxine responsiveness is uncommon, with most genetically confirmed patients requiring PLP for adequate seizure control. This is highlighted by a meta‐analysis of a cohort of 38 cases, in which only 10% of patients showed a sustained long‐term response to pyridoxine as opposed to the remaining 90% who responded to PLP [3]. Long‐term outcomes of PNPO deficient patients remain suboptimal with some affected individuals showing moderate to severe developmental delay [4]. With early and optimal treatment, individuals with PNPO deficiency can have excellent seizure control and a normal neurodevelopmental outcome [5].

PNPO deficiency is also often associated with prematurity [4, 6], and in some pregnancies, intrauterine seizures have been reported [1], suggesting an antenatal affection. Therefore, prenatal treatment has raised much interest. Also, the delay in treatment initiation after birth is one major prognostic factor in this pathology [7]. Prenatal diagnosis can provide the opportunity of an early management, including in utero, peripartum, and immediate postnatal adapted treatment. Indeed, prenatal pyridoxine supplementation is possible since maternal heterozygosity allows for effective conversion of pyridoxine to active PLP in utero. However, the transition to PLP for peripartum and postnatal treatment is mandatory due to PNPO deficiency.

Three children with PNPO deficiency whose mothers received a multivitamin preparation during pregnancy have been reported [5]. PLP treatment was initiated at birth for case 2 regarding his family history, and cases 3 and 4 had PLP initiated at 2 and 1 months of age respectively, following the onset of seizures. All had a normal neurodevelopmental outcome at 2.5 (Case 2), 7.5 (Case 3), and 11 (Case 4), respectively [5]. Pregnancy multivitamin preparations most frequently contain approximately 10 mg of pyridoxine, 2 mg of riboflavin, and 400 μg of folic acid.

In this article we report 2 new cases of in utero pyridoxine treatment. Both patients had a sibling with PNPO deficiency and were therefore at risk (25%) of PNPO deficiency. The first patient had prenatal testing in regard to his family context and had a positive diagnosis, and shows normal psychomotor development at the age of 10 years. For the second one, there was no prenatal testing. Postnatal molecular testing confirmed that the baby was unaffected. We discuss the potential benefits and the practical modalities of in utero treatment with pyridoxine.

## 2 Case Reports

### 2.1 Case 1

The patient is a 10‐year‐old boy who was diagnosed with PNPO deficiency through prenatal molecular testing due to his family history. His older sister had been diagnosed with PNPO deficiency with a homozygous c.364‐1G>C splice variant in the *PNPO* gene after she presented with neonatal severe epileptic encephalopathy, responsive to PLP. She is currently aged 12 years, and exhibits a severe encephalopathy with intellectual disability and spastic tetraparesis. Both parents were heterozygous carriers. Following the confirmatory prenatal result at 5 months of gestation, the mother received nutritional prophylaxis via a daily multivitamin supplement (Femibion Metafolin 1 tablet/day) containing pyridoxine 1.9 mg (140% RDA [Recommended Dietary Allowance]), folic acid 800 μg (400% RDA) and riboflavin 1.4 mg (140% RDA), which is the cofactor for PNPO. While this dosage does not constitute a high‐dose therapeutic intrauterine treatment, it ensured maternal and fetal vitamin levels exceeding the Recommended Dietary Allowance. The effects of PLP supplementation during pregnancy remain unknown, whereas pyridoxine has been widely used in pregnant women, either as part of multivitamin supplementation or for antiemetic treatment, with a well‐established safety profile [8, 9, 10]. Given that the mother can efficiently metabolize pyridoxine into PLP, the use of pyridoxine during pregnancy is therefore preferable. Here, there were no clinical signs suggestive of intrauterine seizures, and the mother received 50 mg of pyridoxal phosphate at the beginning of labor in order to prevent neonatal seizures. The patient was born at term, and immediately received PLP 40 mg/kg/d in four divided dosages. He was eutrophic at birth and had a good adaptation to extrauterine life, clinical exam was normal. An EEG was performed on the first day of life and showed no sign of seizure activity. Feeding was given without any restriction and PLP treatment was continued.

Regular follow‐up visits were scheduled. The patient exhibited normal psychomotor development during the first months of life, with no seizure episodes. PLP was regularly adapted to weight, around 50 mg/kg/day.

At 1 year of age, hepatomegaly was first noticed, and liver enzymes were elevated (ALAT 168 IU/L [*N* < 45]; GGT 44 IU/L [*N* < 25]; normal bilirubin). Clotting factors were low (Prothrombin time ratio 56% [*N* > 70%]) but with normal factor V suggesting vitamin K deficiency. A liver ultrasound performed at 15 months of age showed increased hepatic echogenicity, with liver size at the upper limit of normal, consistent with stage I fibrosis. Liver enzyme levels decreased but remained moderately elevated (ASAT 75 IU/L [*N* < 60]; ALAT 49 IU/L [*N* < 45]; GGT 41 IU/L [*N* < 25]) and liver function normalized (normal clotting factors). PLP was at that time 50 mg/kg/day.

Parents reported occasional tremors before treatment administration or when daily dosage was below 50 mg/kg/day. A first seizure episode occurred at age 16 months with status epilepticus that resolved after oral midazolam administration. PLP was regularly readjusted to 50 mg/kg/d, and pyridoxine (250 mg/day) and folinic acid (10 mg/day) were added. The patient continued to show progress and had normal psychomotor development. Liver laboratory parameters showed elevated enzymes (ASAT 139 IU/L [*N* < 60] ALAT 123 IU/L [*N* < 49]; GGT 79 IU/L [*N* < 25]) and hepatomegaly was still present. Alpha‐fetoprotein was also mildly increased at 20 ng/mL (*N* < 12).

Seizure episodes were rare and rapidly resolved after midazolam administration. PLP dose was maintained around 50 mg/kg/day. At age 6 years, he still had increased liver enzymes (ALAT 76 IU/L [*N* < 30]; GGT 135 IU/L [*N* < 16]) with normal hepatocellular function (normal clotting factors). Liver ultrasound was performed and showed a liver slightly enlarged, hyperechogenic and finely heterogenous with a diffuse micronodular appearance. Liver elastography was measured at 17.4 kPa (stage III fibrosis) and liver attenuation was measured at 0.72 dB/cm/MHz (grade I steatosis). This was presumably ascribed to PLP liver toxicity, and therefore dose was slowly decreased to 40 mg/kg/day, with no new seizure episodes.

This decrease allowed normalization of alpha‐fetoprotein within a few months. Liver enzymes still showed fluctuating yet moderate elevation (not shown). Liver MRI was performed 6 months after (7 years old), and appeared to show a reduction in signs of liver involvement, with a slight hepatomegaly, but no steatosis nor nodules were found. A control ultrasound was performed 1 year later (8.5 years old), the liver was of normal size with regular contours and a homogenous echostructure. An extensive workup was performed to rule out another cause of liver disease (not shown). Negative results supported the conclusion that his liver abnormalities could be explained by liver toxicity of PLP.

At 10 years of age, the patient demonstrates normal psychomotor development, attends school without difficulties, and shows no abnormalities on physical examination. Seizures occur only rarely, and PLP therapy is maintained at the lowest effective dose, currently 40 mg/kg/day. A new liver MRI was performed and confirmed the previous improvement.

**FIGURE 1:**
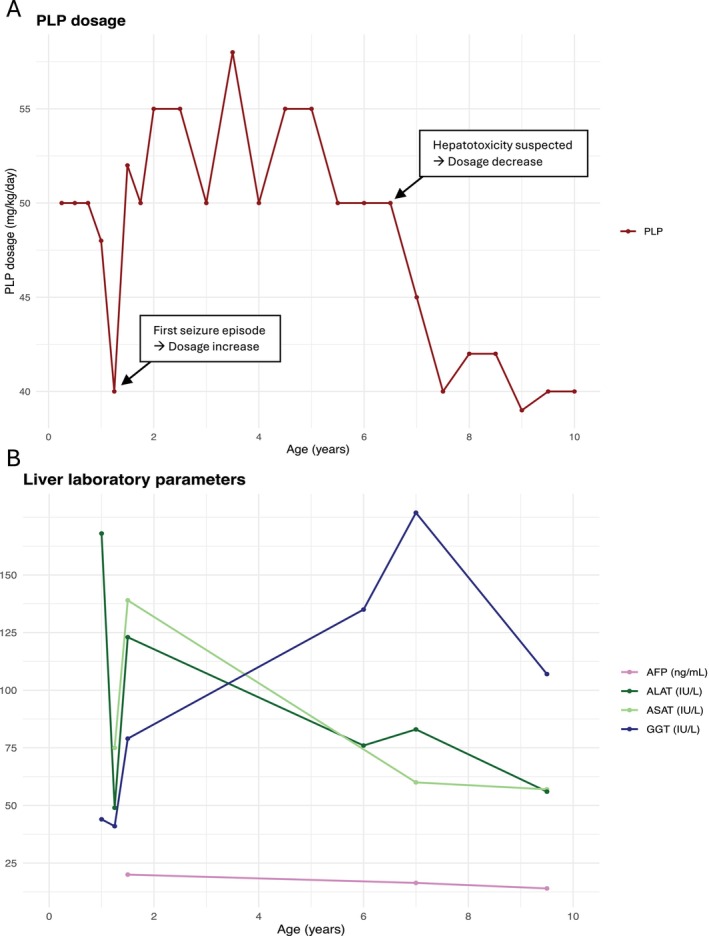
(A) Evolution of PLP dosage in Case 1. (B) Liver parameters evolution with time.

PLP dosage and liver enzymes for this case are summarized in Figure 1.

### 2.2 Case 2

Prenatal testing was offered due to the family history of PNPO deficiency, including an affected sibling, but was declined. During the pregnancy, the mother received exclusive pyridoxine supplementation (Magnevie B6, containing 10 mg pyridoxine, 770% of the RDA). At birth, the newborn was immediately treated with PLP at 40 mg/kg/day divided into four doses, pending molecular results. Ten days later, testing confirmed the child was unaffected. PLP was then gradually withdrawn over 3 days, and the baby remained asymptomatic.

## 3 Discussion

The clinical course in Case 1 underscores the therapeutic benefit of in utero pyridoxine supplementation, which successfully prevented the onset of both in utero and postnatal seizures and allowed optimal neurodevelopment. Despite this early control, postnatal adaptation of PLP dosage was necessary following the occurrence of subsequent seizures.

Pyridoxine supplementation during pregnancy was previously reported in three children [5]. The first mother (Case 2) received 2.6 mg/day of pyridoxine and an additional PLP supplement before delivery in the context of an affected sibling. Mothers of Cases 3 and 4 received pyridoxine as part of a multivitamin supplementation during pregnancy (dose not specified). At birth, Case 2 was started on PLP at a dose of 30 mg/kg/day. The patient had no seizures in the neonatal period and showed a normal clinical neurodevelopment at the age of 2.5 years. Case 3 had no neonatal specific treatment and had a first seizure episode at age 4 weeks, which was resistant to regular antiepileptic drugs (phenobarbitone, phenytoin and oxcarbazepine) and pyridoxine. PLP treatment was initiated at 8 weeks and allowed cessation of seizures and EEG normalization. However, normal neurodevelopment was described at age 7.5 years. In Case 4, different seizure types appeared during the first days of life, also resistant to anticonvulsants and pyridoxine, and PLP was initiated at 28 days, allowing seizure resolution. He also presented a normal neurological examination at the age of 11 years.

More reports are needed but these patients suggest that a modest supplement of B6 such as is in a normal pregnancy multivitamin supplement can be sufficient to ensure a good outcome for a fetus with PNPO deficiency at least in some cases, especially by preventing seizures during prenatal and neonatal periods. The widespread use of these supplements without reports of side effects suggests that there should be no adverse effects on mother or fetus.

Starting PLP treatment for PNPO deficiency at birth at a dose of 30 mg/kg/day can prevent neonatal seizures, especially when an affected sibling had been responsive to PLP rather than pyridoxine. Nevertheless, seizures can be observed ultimately after a few months. In our patient (Case 1), regular adaptation of PLP dosage ultimately achieved effective long‐term control of seizures and resulted in highly satisfactory psychomotor development in spite of the occasional occurrence of seizures.

Nevertheless, PLP treatment was associated with a meaningful risk of hepatotoxicity that seems to be dose dependent as previously suggested [11, 12, 13]. Cases described in the literature include significant elevations in liver enzymes that improved with PLP dose reduction, as well as biopsy‐confirmed cirrhosis following prolonged therapy at doses up to 100 mg/kg/day [12]. There are also reports linking long‐term PLP treatment to severe outcomes such as hepatocellular carcinoma in adolescence, highlighting the potential severity of liver complications [11, 14].

In our case 1, besides the improvement of liver enzymes after PLP dose decrease, we observed reversible liver lesions such as micronodules. Managing therapy therefore requires balancing clinical efficacy with hepatic tolerance, and mandates regular monitoring of liver function tests, but also regular ultrasound monitoring, especially with elevated alpha‐fetoprotein.

Last, while prenatal pyridoxine supplementation seems to be both safe and highly beneficial when a diagnosis of PNPO deficiency is suspected, postnatal management requires more caution. Ideally, treatment should be guided by molecular confirmation. In fact, prophylactic pyridoxine supplementation of neonates at risk for pyridoxine‐dependent epilepsy may lead to neuronal hyperexcitability in those who are ultimately unaffected [15], especially given the high doses of pyridoxine and PLP required to manage these severe forms of epilepsy. Immediate postpartum biochemical and molecular work‐up is therefore crucial.

Case 2 illustrates and confirms the safety of in utero pyridoxine supplementation even in a fetus ultimately shown to be unaffected. The child received prenatal pyridoxine and immediate postnatal PLP, which was gradually withdrawn after the negative molecular result. The absence of adverse events during pregnancy or after birth confirms that modest pyridoxine supplementation during gestation is safe for both mother and fetus, and that PLP treatment at birth does not appear to be associated with neuronal hyperexcitability, providing reassurance for at‐risk pregnancies when PNPO deficiency cannot be confirmed prenatally.

## 4 Conclusion

Normal neurodevelopmental outcome after early PLP supplementation in PNPO deficiency was previously reported [5]. Our patient, now 10 years old, represents one of the longest follow‐ups reported in a PNPO patient treated with PLP from birth. While no causal link can be made from two single observations, the absence of maternal or fetal adverse effects during prenatal pyridoxine and riboflavin supplementation provides additional clinical reassurance regarding the feasibility of this approach. Riboflavin supplementation may theoretically enhance residual PNPO activity through its role as an FMN cofactor. Overall, this case supports early diagnosis and prompt PLP initiation and contributes further clinical data regarding prenatal management strategies in PNPO deficiency.

## Author Contributions

C.P. participated in the acquisition of data, drafting and editing of the manuscript. M.S. participated in the supervision of the project, review and edition of the manuscript. S.P., M.G., J.D., L.F., A.I., A.B., J.‐F.B., P.B.M., P.T.C., and M.S. participated in the critical review of the manuscript and approved it for publication.

## Funding

The authors have nothing to report.

## Ethics Statement

This study has been carried out in accordance with The Code of Ethics of the World Medical Association (Declaration of Helsinki).

## Consent

Written informed consent was obtained from the parents of each patient prior to genetic testing. Parents gave their consent for publication.

## Conflicts of Interest

The authors declare no conflicts of interest.

## Data Availability

The data that support the findings of this study are available from the corresponding author upon reasonable request.
